# Deaths and adverse events from adjuvant therapy with immune checkpoint inhibitors in solid malignant tumors: A systematic review and network meta‐analysis

**DOI:** 10.1002/cai2.34

**Published:** 2022-11-14

**Authors:** Ruiyang Xie, Jie Wu, Bingqing Shang, Xingang Bi, Chuanzhen Cao, Youyan Guan, Hongzhe Shi, Jianzhong Shou

**Affiliations:** ^1^ Department of Urology, National Cancer Center/National Clinical Research Center for Cancer/Cancer Hospital Chinese Academy of Medical Sciences and Peking Union Medical College Beijing China

**Keywords:** immune checkpoint inhibitor, adjuvant therapy, cancer, adverse event, death

## Abstract

**Background:**

By prolonging overall survival and reducing disease recurrence rates, immune checkpoint inhibitors (ICIs) are an emerging adjuvant therapy option for patients with resectable malignant tumors. However, the safety profile (deaths and adverse events [AEs]) of adjuvant ICIs has not been fully described.

**Methods:**

We searched the literature for phase III randomized clinical trials that compared PD‐1, PD‐L1, and CTLA‐4 inhibitors in solid malignant tumors. Incidences of death, discontinuation, AEs of any cause, treatment‐related adverse events (TRAEs), and immune‐related adverse events (IRAEs) were extracted for the network meta‐analysis. Network meta‐analyses with low incidence and poor convergence are reported as incidences with 95% confidence intervals (95% CIs).

**Results:**

Ten randomized clinical trials that included 9243 patients who received ICI adjuvant therapy were eligible. In total, 21 deaths due to TRAEs were recorded, with an overall incidence of 0.40% (95% CI: 0.26–0.61). The treatment‐related mortality rates for ipilimumab (0.76%, 95% CI: 0.31–1.55) and atezolizumab (0.56%, 95% CI: 0.18–1.31) were higher than for pembrolizumab (0.24%, 95% CI: 0.10–0.56) and nivolumab (0.30%, 95% CI: 0.08–0.77). The most frequent causes of death were associated with the gastrointestinal (0.10%, 95% CI: 0.04–0.24) and pulmonary (0.08%, 95% CI: 0.03–0.21) systems. Compared with the control arm, we found that nivolumab (odds ratio [OR]: 2.73, 95% CI: 0.49–15.85) and atezolizumab (OR: 12.43, 95% CI: 2.42–78.48) caused the fewest grade ≥3 TRAEs and IRAEs. Commonly reported IRAEs of special interest were analyzed, and two agents were found to have IRAEs with incidences >10%, i.e., hepatitis for atezolizumab (14.80%, 95% CI: 12.53–17.32) and hypophysitis for ipilimumab (13.53%, 95% CI: 11.38–15.90).

**Conclusions:**

Ipilimumab and atezolizumab were correlated with higher treatment‐related death rates than pembrolizumab and nivolumab, in which the gastrointestinal and pulmonary systems were mostly involved. Regarding severe TRAEs and IRAEs, nivolumab and atezolizumab are likely to be the safest agent, respectively. This study will guide clinical practice for ICI adjuvant therapies.

## Graphical abstract

Immune checkpoint inhibitors (ICIs) are emerging as adjuvant therapy for patients with high‐risk resectable malignant tumors, by prolonging overall survival and reducing disease recurrence. The present study provides the safety profile of deaths and adverse events in four approved ICIs. Comprehensively, nivolumab and atezolizumab were likely to be the safest agents.

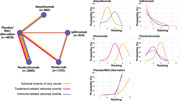

**Abbreviations**
AE: adverse event
CRS: cytokine release syndrome
DFS: disease‐free survival
ICI: immune checkpoint inhibitor
IRAE: immune‐related adverse event
OR: odds ratio
OS: overall survival
RCT: randomized clinical trial
TRAE: treatment‐related adverse event

## 1 INTRODUCTION

Immune checkpoint inhibitors (ICIs) have revolutionized the therapeutic paradigm of cancer. Inhibitors of PD‐1, PD‐L1, and CTLA‐4 have been approved by the European Medicines Agency and the US Food and Drug Administration for locally advanced and metastatic malignancies [1, 2]. Several clinical trials explored the role of ICIs in the adjuvant setting and showed encouraging results [3, 4]. Compared with chemotherapy and targeted therapy, ICIs prolonged overall survival (OS) and disease‐free survival (DFS); however, they can also cause autoimmune‐like disorders and even life‐threatening adverse events (AEs) [5]. Several systematic reviews have investigated the incidences of treatment‐related adverse events (TRAEs) in patients who received ICIs [6, 7]. Most studies included in these previous meta‐analyses focused on AEs in patients with locally advanced or metastatic cancer. However, toxicity and tolerance are important in the treatment decisions of high‐risk patients with localized disease. Treatment‐related deaths from adjuvant immunotherapy are striking for patients with the potential for a long life expectancy.

To date, the CTLA‐4 inhibitor ipilimumab, the PD‐L1 inhibitor atezolizumab, and the PD‐1 inhibitors pembrolizumab and nivolumab have shown positive DFS results as adjuvant monotherapies [4, 8, 9, 10]. The results of the EORTC 18071 trial further suggested that adjuvant ICI monotherapy conferred significantly prolonged OS in melanoma patients [10]. Notably, several major guidelines recommend adjuvant ICI monotherapy in the treatment of solid malignancies without conclusive evidence of improved OS. Thus, understanding the toxicological profiles and treatment‐related deaths associated with ICIs in the perioperative setting is critical for clinical practice.

We performed this systematic review and network meta‐analysis to determine the toxicity outcomes, especially treatment‐related deaths, of adjuvant immunotherapy. We summarized TRAEs with ICI‐based adjuvant monotherapy in published phase III clinical trials and investigated the different incidences of severe AEs among the ICIs.

## 2 METHODS

### 2.1 Search methods and study selection

This systematic review and network meta‐analysis were conducted following the Preferred Reporting Items for Systematic Reviews and Meta‐Analyses (PRISMA) statement (Supporting Information: Table S1) [11]. The protocol was registered in the Prospective Register of Systematic Reviews (PROSPERO CRD42022339062). We searched the Cochrane, Embase, and PubMed databases for relevant studies by using the following search terms: (“PD‐1” OR “PD‐L1” OR “CTLA‐4”) AND (“phase III” OR “phase 3”) AND “adjuvant therapy”. Papers published as of May 20, 2022 were included. Relevant papers from the references of identified studies were also examined. The full search criteria are presented in Supporting Information: Table S5.

Two independent reviewers (R. Xie and J. Wu) performed the study screening. Disagreements were resolved by a third reviewer (J. Shou). The inclusion criteria for trial selection were as follows: (1) phase III randomized clinical trials (RCTs) in which participants received adjuvant PD‐1, PD‐L1, or CTLA4 inhibitor monotherapy; (2) the patients then received tumor resection or radiotherapy; (3) tabulated data on TRAEs and deaths were available; and (4) published in English. Studies that did not satisfy the inclusion criteria were excluded. Other exclusion criteria were as follows: (1) data from conferences before 2022; (2) articles in which participants were treated with sequential monotherapies; and (3) trials in which treatments other than ICIs were involved. The full literature was retrieved for further qualitative review.

### 2.2 Data extraction

General characteristics including start year, study ID, patient age, sample size, intervention arm, patient sex, and the control arm were extracted. Data for reported AEs (AEs of any cause, TRAEs, and immune‐related adverse events [IRAEs]) and deaths (death due to AEs and deaths due to TRAEs) were extracted. AEs in all‐grade and grade ≥3 were defined as grades 1–5 and 3–5, respectively. Any noted unintentional or unfavorable clinical signs or symptoms, including complications of miscarriage in all‐causes were denoted as AEs [12]. TRAEs are any AEs that in the investigators’ opinion may have been caused by the study medication with reasonable possibility. IRAEs are autoimmune conditions caused by unintended effects of the ICI‐mediated activation of the immune system and may occur in any organ system [13]. The safety profiles of the most commonly reported all‐grade AEs were also analyzed for categorization on the basis of organ systems: renal, hepatic, pulmonary, gastrointestinal, skin, endocrine, cardiac, and blood/lymphatic. All AEs were classified in accordance with the Common Terminology Criteria for Adverse Events (CTCAE) version 5.0 [14]. Discontinuation events due to TRAE were also extracted. The risk of bias in each trial was assessed using the Cochrane Risk of Bias Tool and was annotated as high, unclear, or low risk of bias [15]. The following categories were scored: allocation concealment, random sequence generation, blinding of participants and personnel, incomplete outcome data, blinding of outcome assessment, selective reporting, and other biases (Supporting Information: Figure S3). Any disagreements in the study selection, data extraction, and quality assessment were resolved by discussion to achieve a consensus.

### 2.3 Data synthesis and statistical analysis

Odds ratios (ORs) with 95% confidence intervals (95% CIs) are used to describe AE rates. If significant heterogeneity existed, network meta‐analyses of AEs were performed with the Bayesian random‐effects consistency model, which is a conservative approach to dealing with between‐study heterogeneity; otherwise, we used the fixed effects model [16]. Bayesian network modeling confers the advantage of accommodating complex situations by offering a straightforward method for probabilistic statements and predictions on the treatment effects [17]. We then used inconsistency model analysis to show the inconsistency of evidence [18]. The ranking probability of the treatments for AEs was calculated by the surface under the cumulative ranking curve (SUCRA) analysis [17]. Heterogeneity among trials was assessed by the *I* ^2^ values of the consistency model if more than one trial existed. *Ι* ^2^ values greater than 25%, 50%, and 75% indicated low, moderate, and high heterogeneity, respectively [19]. The summary of incidence was calculated by the ratio of events that occurred and total patients. Network meta‐analyses with low incidence and poor convergence were removed and reported as incidence with 95% CIs. The 95% CIs were estimated together with the incidence through binomial probability. We assessed the study inclusion reliability by calculating the estimated sample size (Supporting Information: Table S4) [20]. All of the enrolled studies had large enough sample sizes except for the comparison of nivolumab and ipilimumab (2259 enrolled, 3529 required). However, all published phase III trials to date with valid data for ICI adjuvant therapy were enrolled in this network meta‐analysis (9243 patients in total).

To illustrate the sample size and number of trials, we used the “rjags” and “GeMtc” packages of R 4.0.2 (https://www.r-project.org/) to generate the Bayesian network modeling of AEs [21]. Incidence rates with 95% CIs were calculated with the binconf() function from the “Hmisc” package. Analyses of heterogeneity and ranking probability were also run in R. To further determine the heterogeneity effects, the number of adaptations was set to 5000, and the sample iteration parameter was adjusted to 20,000.

## 3 RESULTS

### 3.1 Characteristics of the enrolled trials and systematic review

**Figure 1:**
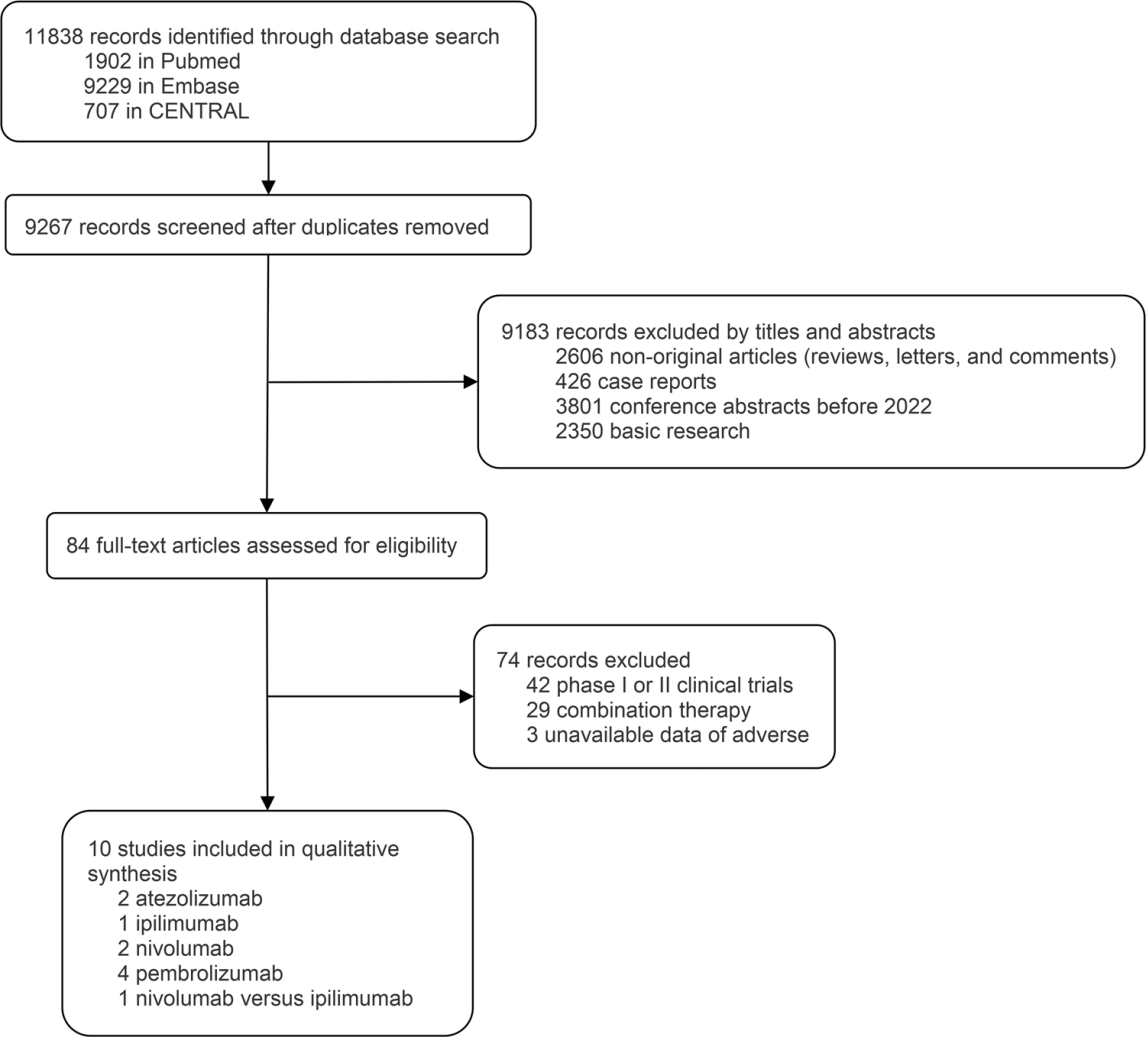
Study selection

In total, 9267 records from the database were identified and screened for titles and abstracts (Figure 1). Ten phase III RCTs were included in the analysis [3, 4, 8, 9, 10, 22, 23, 24, 25, 26]. To ensure the reliability of reported results and quality control, phase II clinical trials were not included in the study. The ICIs are identified as PD‐1 inhibitors (pembrolizumab and nivolumab), PD‐L1 inhibitor (atezolizumab), and CTLA‐4 inhibitor (ipilimumab). Among the 10 studies encompassing 9243 patients, 7 reported deaths due to AEs, 10 reported deaths due to TRAEs, 10 reported AEs of any cause, 8 reported TRAEs, 6 reported IRAEs, and 6 reported discontinuations due to TRAEs. The general characteristics of these studies, including the group interventions and patient populations for safety assessment, are summarized in Table 1. The efficacy of ICIs (DFS and OS) is summarized in Supporting Information: Table S6. All of the enrolled studies showed a survival benefit of ICIs except for the IMvigor010 study.

**Table 1:** Studies that evaluated the safety of adjuvant immune checkpoint inhibitor monotherapy

	Study	Start year	Study objective	ICI regimen (arm1/arm2)	No. of patients in safety assessment	Median age	Sex of male
1	EORTC 18071 (NCT00636168)	2008	Melanoma	Ipilimumab/placebo	471/474	51/52	296/293
2	IMvigor010 (NCT02450331)	2015	Muscle‐invasive urothelial carcinoma	Atezolizumab/observation	390/397	67/66	322/316
3	KEYNOTE‐091 (NCT02504372)	2015	Non‐small‐cell lung cancer	Pembrolizumab/placebo	580/581	65/65	401/403
4	KEYNOTE‐054 (NCT02362594)	2015	Melanoma	Pembrolizumab/placebo	509/502	54/54	324/304
5	IMpower010 (NCT02486718)	2015	Non‐small‐cell lung cancer	Atezolizumab/BSC	495/495	62/62	337/335
6	CheckMate 238 (NCT02388906)	2015	Melanoma	Nivolumab/ipilimumab	452/453	56/54	258/269
7	CheckMate 577 (NCT02743494)	2016	Esophageal or gastroesophageal junction cancer	Nivolumab/placebo	532/260	62/61	449/222
8	CheckMate 274 (NCT02632409)	2016	Muscle‐invasive urothelial carcinoma	Nivolumab/placebo	351/348	65/66	265/275
9	KEYNOTE‐564 (NCT03142334)	2017	Renal cell carcinoma	Pembrolizumab/placebo	488/496	60/60	347/359
10	KEYNOTE‐716 (NCT03553836)	2018	Melanoma	Pembrolizumab/placebo	483/486	60/61	300/289

### 3.2 Overall incidence of deaths caused by ICI adjuvant therapy

**Figure 2:**
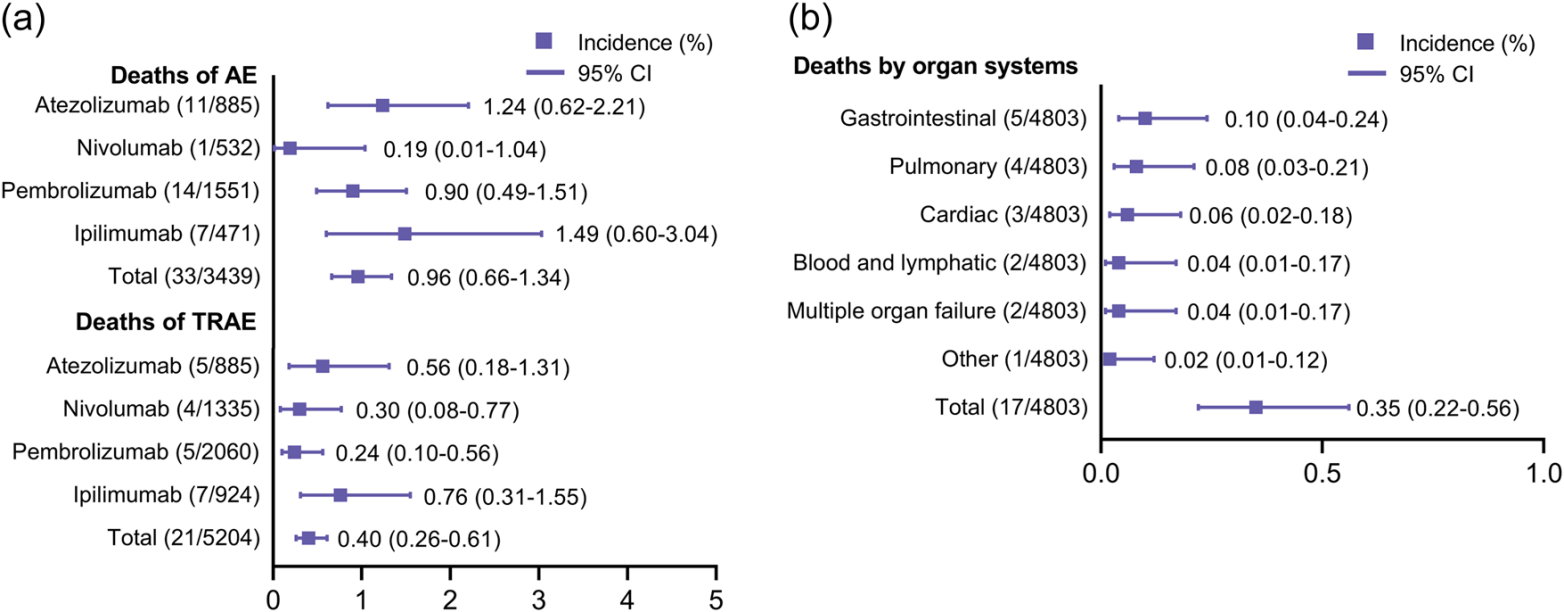
Incidences of deaths in clinical trials of immune checkpoint inhibitors as adjuvant therapy. (a) Incidences of deaths due to adverse events (AEs) of any cause and treatment‐related adverse events (TRAEs). (b) Incidences of deaths categorized by organ systems.

To fully describe the landscape of death events, we separately calculated the incidences of deaths due to AEs and TRAEs (Figure 2a). Among the 33 death events reported in the seven trials, the death rate of AEs of any cause was 0.96% (95% CI: 0.66–1.34). Patients who received adjuvant ipilimumab (1.49%, 95% CI: 0.60–3.04) and atezolizumab (1.24%, 95% CI: 0.62–2.21) had overall mortality rates higher than 1%. However, three enrolled studies (KEYNOTE‐054, CheckMate 274, and CheckMate 238) did not report all‐cause death events. We further investigated the death events due to TRAEs, of which 21 death events were noted, with an overall incidence of 0.40% (95% CI: 0.26–0.61). In contrast to other ICIs, pembrolizumab caused the fewest treatment‐related deaths (0.24%, 95% CI: 0.10–0.56), whereas treatment‐related death rates of both ipilimumab (0.76%, 95% CI: 0.31–1.55) and atezolizumab (0.56%, 95% CI: 0.18–1.31) were higher than the average. The causes of death for patients treated with ICI adjuvant therapy were complex. All deaths due to TRAEs are summarized in Table 2. The KEYNOTE‐716 and KEYNOTE‐564 studies conferred no treatment‐related deaths. In total, 17 deaths with detailed causes were extracted and noted, among which colitis was prominent in ipilimumab, with four cases of death. Despite the incomplete description of relevant causes of death in the KEYNOTE‐091 study, the current profile suggests that pembrolizumab caused only one death event from myositis in the KEYNOTE‐054 study. Next, we categorized the 17 death events with reported causes on the basis of organ systems. The most frequent causes of death by organ system were gastrointestinal (0.10%, 95% CI: 0.04–0.24), followed by pulmonary (0.08%, 95% CI: 0.03–0.21), cardiac (0.06%, 95% CI: 0.02–0.18), and blood/lymphatic and multiple organ failure (0.04%, 95% CI: 0.01–0.17; Figure 2b). There was one treatment‐related death from myositis that was classified as “other.”

**Table 2:** Summary of deaths due to treatment‐related adverse events

Drugs	Cause of TRAE death	Number	Study
Pembrolizumab	Myositis	1	KEYNOTE‐054
Nivolumab	Pneumonitis	2	CheckMate 274
	Bowel perforation	1	CheckMate 274
	Cardiac arrest	1	CheckMate 577
Atezolizumab	Interstitial lung disease	1	IMpower010
	Multiple organ dysfunction syndrome	1	IMpower010
	Myocarditis	1	IMpower010
	Acute myeloid leukemia	1	IMpower010
	Acute respiratory distress syndrome	1	IMvigor010
Ipilimumab	Marrow aplasia	1	CheckMate 238
	Colitis	4	CheckMate 238; EORTC 18071
	Myocarditis	1	EORTC 18071
	Multiorgan failure with Guillain‐Barre syndrome	1	EORTC 18071
**Total**		**17**	

Abbreviation: TRAE, treatment‐related adverse event.

### 3.3 Network meta‐analysis with the consistency model

**Figure 3:**
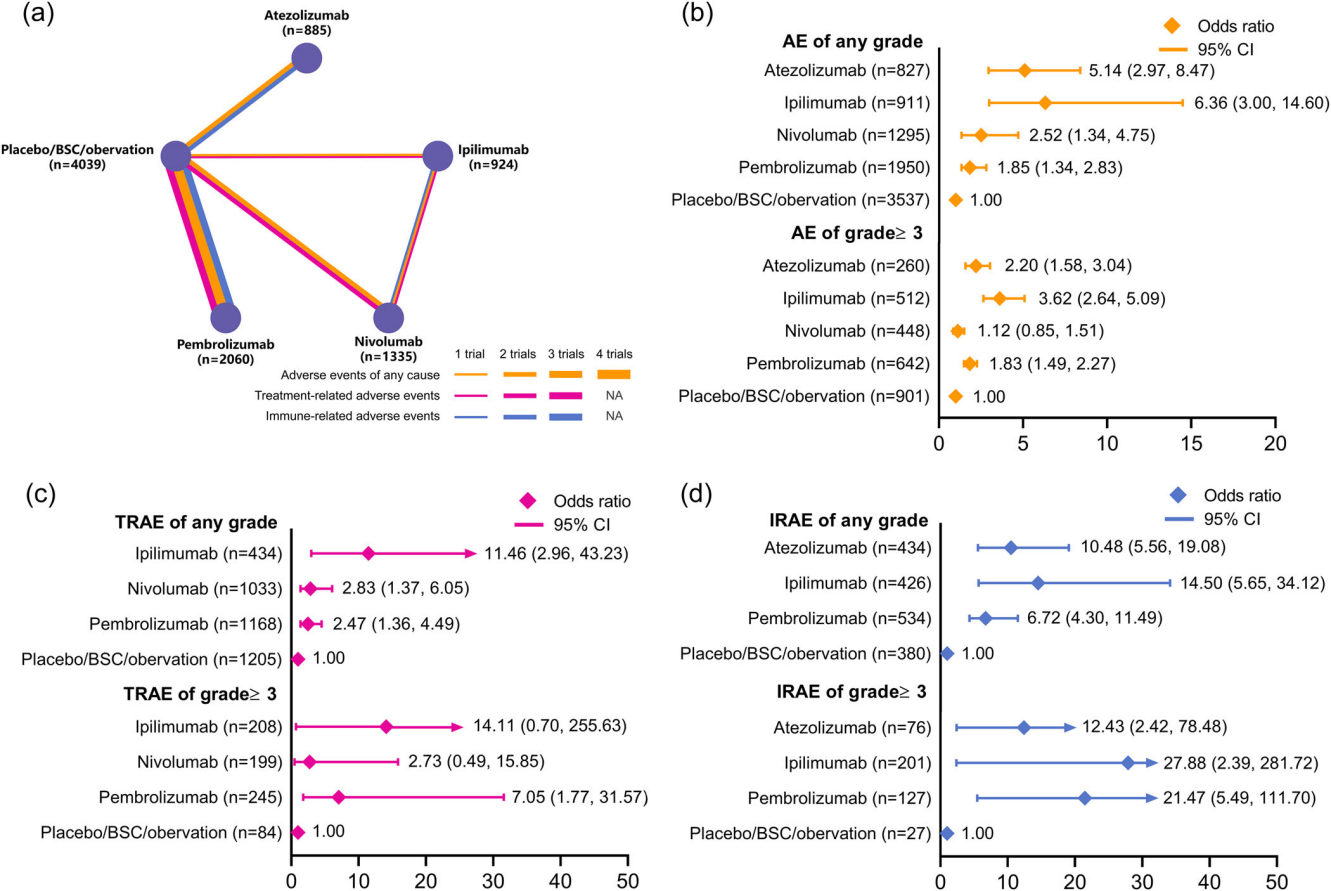
Network plot and pooled estimates of comparisons of all‐cause adverse events (AEs) from immune checkpoint inhibitors as adjuvant therapy. (a) Comparisons of any cause AEs, treatment‐related adverse events (TRAEs), and immune‐related adverse events (IRAEs). Each node represents a treatment, and each line represents a type of head‐to‐head comparison. The total number of patients enrolled in the safety assessments are shown in brackets. The width of lines is proportional to the number of trials comparing the connected treatments. (b) Odds ratios (ORs) with 95% confidence intervals (95% CIs) for any cause AEs. (c) ORs with 95% CIs for TRAEs. (d) ORs with 95% CI for IRAEs. The numbers in brackets of the forest plots show the numbers of patients in which the AEs occurred.

Figure 3a shows the network plots for AEs of different causes from the 10 studies of the four ICI treatments. The arms of placebo, best supportive care, and observation were stratified into a control arm due to not receiving any treatment interventions. As shown in Figure 3b, the ORs of ipilimumab versus the control arm were 6.36 (95% CI: 3.00–14.60) and 3.62 (95% CI: 2.64–5.09) for AEs of any cause in all‐grades and of grade ≥3, respectively. Notably, pembrolizumab had similar ORs when compared with the control arm in these two comparisons (OR: 1.85, 95% CI: 1.34–2.83 and OR: 1.83, 95% CI 1.49–2.27). Regarding TRAEs, we found that ipilimumab had high ORs in any grade (OR: 11.46, 95% CI: 2.96–43.23) and grade ≥3 (OR: 14.11, 95% CI: 0.70–255.63), whereas nivolumab caused much fewer TRAEs in both categories (any grade OR: 2.83, 95% CI: 1.37–6.05 and grade ≥3 OR: 2.73, 95% CI: 0.49–15.85). In terms of grade ≥3 IRAEs, the OR of atezolizumab versus the control arm (OR: 12.43, 95% CI: 2.42–78.48) was significantly lower than those of the other ICIs. The ranking probability for each ICI is presented in Supporting Information: Figure S1. The consistency analysis of discontinuations due to AEs indicated that ipilimumab had the most discontinuation events (OR: 20.52, 95% CI: 6.68–58.18; Supporting Information: Figure S2a). Supporting Information: Figure S2b shows the incidences of discontinuation for all ICIs, in which nivolumab had the fewest discontinuation events (13.26%, 95% CI: 11.55–15.19).

**Figure 4:**
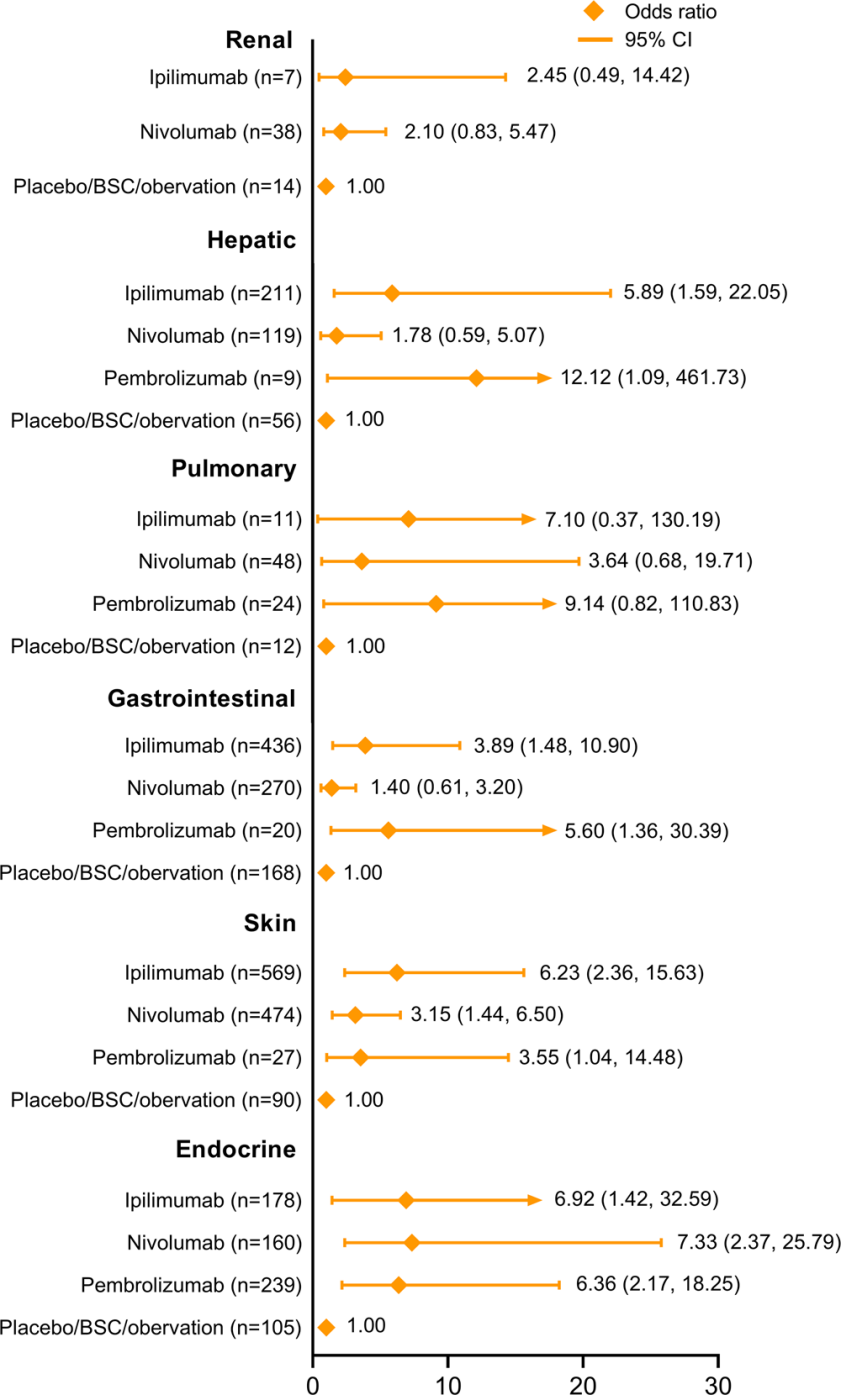
Pooled analysis of all‐cause and all‐grade adverse events (AEs) categorized by organ system. The data summarize reported immune‐related adverse events (IRAEs) from the included trials; treatment‐related adverse events (TRAEs) were included when IRAEs were not recorded. The numbers in brackets of the forest plots show the numbers of patients in which the AEs occurred.

By examining the complete data of the 10 enrolled studies, we found that organ system AEs were reported either as IRAEs or TRAEs in each study. As shown in Figure 4, we analyzed IRAEs or TRAEs of all‐grades on the basis of organ systems. For renal‐ and urinary‐related AEs, only nivolumab and ipilimumab were included, as there were only valid data for these, and they showed ORs versus the control arm of 2.10 (95% CI: 0.83–5.47) and 2.45 (95% CI: 0.49–14.42), respectively. Nivolumab had the lowest OR for hepatic AEs (OR: 1.78, 95% CI: 0.59–5.07), while the OR of pembrolizumab was much higher (OR: 12.12, 95% CI: 1.09–461.73). Regarding pulmonary events, the comparisons indicated nivolumab had the fewest AEs, with an OR of 3.64 (95% CI: 0.68–19.71). Gastrointestinal system AEs from pembrolizumab were notable (OR: 5.60, 95% CI: 1.36–30.39), whereas nivolumab is likely to be the safest agent for gastrointestinal AEs (OR: 1.40, 95% CI: 0.61–3.20). For AEs of the skin, ipilimumab caused more events than the other ICIs (OR: 6.23, 95% CI: 2.36–15.63). Unlike the results for other systems, the analysis of endocrine AEs suggested that nivolumab had the highest OR value compared to the control arm (OR: 7.33, 95% CI: 2.37–25.79).

**Figure 5:**
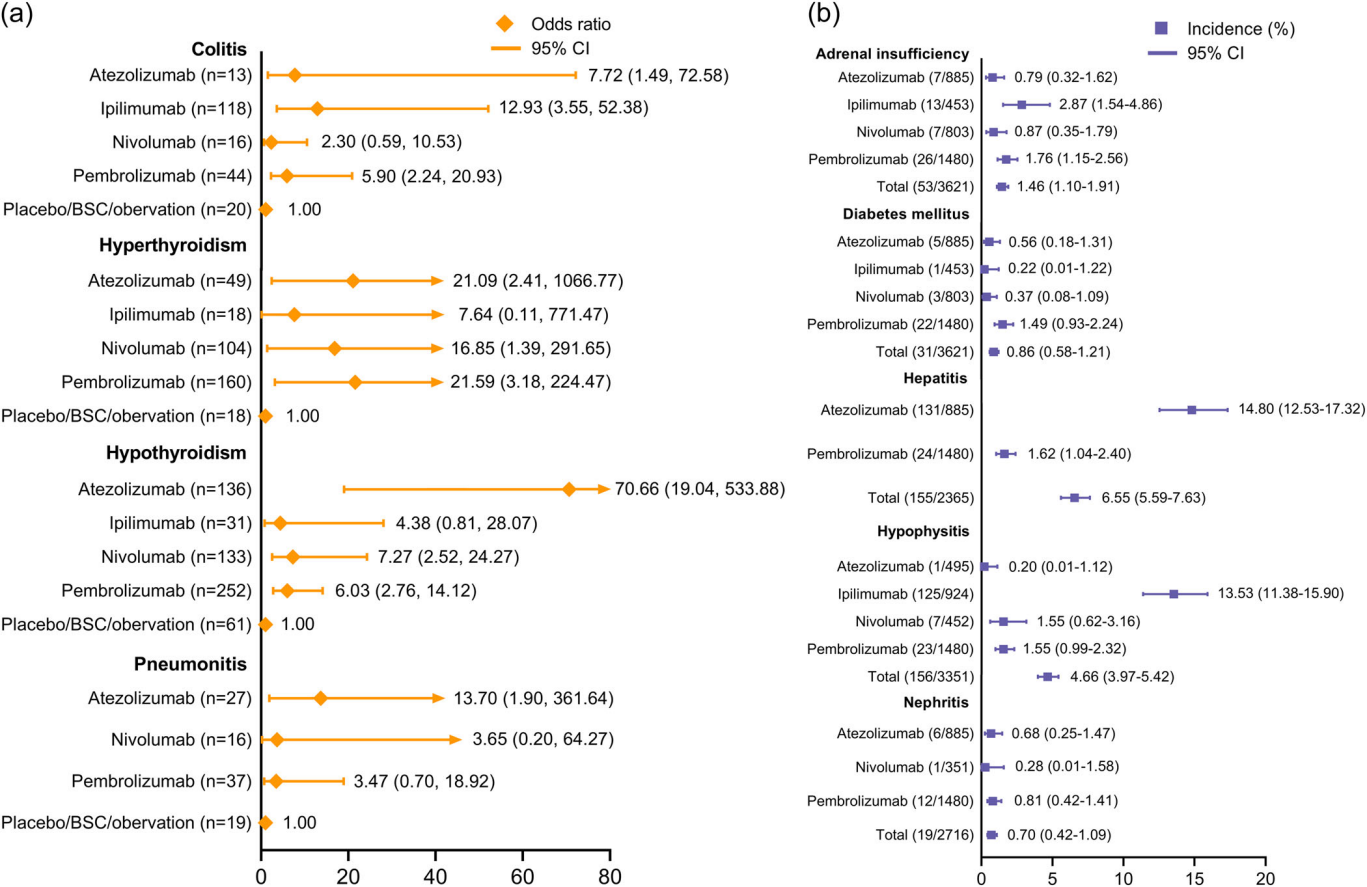
Immune‐related adverse events (IRAEs) of special interest are frequently reported with immune checkpoint inhibitors as adjuvant therapy. (a) Consistency model analyses showing odds ratios (ORs) with 95% confidence intervals (CIs). (b) Incidences of IRAEs of special interest. Network meta‐analyses with poor convergence values are presented as incidences; others are shown in the network meta‐analyses.

IRAEs of special interest that are frequently reported for ICI therapies were also analyzed (Figure 5). The consistency model analyses with convergence values near 1.00 are denoted as ORs (Figure 5a), while other IRAEs are presented as incidence rates with 95% CIs (Figure 5b). For immune‐related colitis, ipilimumab was observed to have the highest OR versus the control arm (OR: 12.93, 95% CI: 3.55–52.38), while nivolumab led to the least risk of colitis (OR: 2.30, 95% CI: 0.59–10.53). In terms of thyroid disorders, pembrolizumab and atezolizumab caused the most hyperthyroidism events (OR: 21.59, 95% CI: 3.18–224.47) and hypothyroidism (OR: 70.66, 95% CI: 19.04–533.88), respectively. Compared with nivolumab and pembrolizumab, atezolizumab had a higher OR for pneumonitis (OR: 13.70, 95% CI: 1.90–361.64). Next, we analyzed incidences of adrenal insufficiency, among which atezolizumab had the least IRAEs (0.79%, 95% CI: 0.32–1.62). For diabetes mellitus, we found that pembrolizumab correlated with the highest incidence rate (1.49%, 95% CI: 0.93–2.24). Notably, there were two agents with incidence rates >10% for specific AEs, that is, hepatitis for atezolizumab (14.80%, 95% CI: 12.53–17.32) and hypophysitis for ipilimumab (13.53%, 95% CI: 11.38–15.90).

### 3.4 Assessments of inconsistency and heterogeneity

The variance of the consistency and inconsistency model are presented in Supporting Information: Table S2. The heterogeneity of general AEs was estimated and shown in Supporting Information: Table S3. The *Ι* ^2^ value suggested moderate heterogeneity in the analysis of TRAEs (*Ι* ^2^ = 68.2 for TRAEs of any grade, *Ι* ^2^ = 73.1 for grade ≥ 3 TRAEs), whereas we found low heterogeneity in all‐cause AEs and IRAEs. Supporting Information: Figure S3 shows the quality assessment, as scored by the Cochrane risk of bias tool. The KEYNOTE‐091 study was reported as a conference abstract, thus the risk of bias in each aspect was beyond moderate. Other studies were assessed to have an acceptable low risk of bias.

## 4 DISCUSSION

This systematic review and network meta‐analysis of ICI adjuvant therapies evaluated 10 phase III RCTs for patients with solid malignancies. We summarized the safety profiles of PD‐1, PD‐L1, and CTLA‐4 inhibitors as monotherapy using the data of four agents (pembrolizumab, nivolumab, atezolizumab, and ipilimumab). The general results indicated the following: (1) the overall incidence of treatment‐related deaths from ICI adjuvant therapy was 0.40%, among which the PD‐1 inhibitors had fewer death events than the PD‐L1 and CTLA‐4 inhibitors; (2) for ICI adjuvant therapy, the most common TRAEs that led to death were associated with the gastrointestinal and pulmonary systems; (3) ipilimumab caused the most all‐cause grade ≥ 3 AEs, while nivolumab had the best safety with regard to grade ≥ 3 AEs and TRAEs; and (4) among the two PD‐1 inhibitors, discontinuation events and AEs of special interest suggested that nivolumab was more likely to be a safe agent than pembrolizumab.

Among patients in clinical trials who undergo tumor resection and receive a full dose of adjuvant therapy under the modified supervision of the investigators, the baseline characteristics of the general conditions are supposed to be tolerable for the upcoming treatments. In real‐world experiences, patients may have more pre‐existing diseases and morbidity than those who are enrolled in clinical trials, possibly leading to a higher rate of deaths and AEs. The aim of adjuvant therapy for cancer is to reduce the occurrence of local recurrence and distant metastasis as well as to prolong OS. Unlike patients with metastatic cancer, surgery can provide considerable recovery benefits for patients with high‐risk localized tumors; however, the potential death risk of further ICI treatments has the possibility of eliminating the acquired benefits of surgical resection. Therefore, the issue of dose safety needs to be considered even more carefully for ICI adjuvant therapy than for the treatment of metastatic lesions.

Deaths caused by AEs and TRAEs are the most severe outcomes in the clinical experience. Before treatment starts, the informed consent should objectively state the previously reported death incidence rates correlated with ICIs in the adjuvant setting. Wang et al. investigated the TRAEs of PD‐1 and PD‐L1 inhibitors mostly in patients with advanced or metastatic disease, and the overall incidence of treatment‐related death was 0.45% [7]. Our study suggested that the incidence of treatment‐related death from ICI adjuvant monotherapy was 0.40%, which was slightly lower than their findings, and we further found that the incidence was 0.32% when only enrolling atezolizumab, nivolumab, and pembrolizumab. However, in this study, the incidence of death from ICI adjuvant therapy was calculated from the accumulated experience of CRTs, and our findings suggested that the prediction and prevention of treatment‐related death remains an emerging issue. When analyzing the causes of death for each treatment, we found that pulmonary‐ and cardiac system‐related deaths were shared among all the ICIs, while the gastrointestinal system was the most prominent cause of treatment‐related death. Previous studies have found that the most commonly reported treatment‐related deaths were associated with the respiratory system [7]. Considering that the lungs are often involved in distant metastasis, the contributions of the malignancy to TRAEs need further exploration. Moreover, ipilimumab was correlated with a higher incidence of lethal gastrointestinal AEs. As reported in a previous study, the onset of immune‐related colitis was more frequent in patients treated with CTLA‐4 inhibitors than in those who received PD‐1/PD‐L1 inhibitors [27, 28]. The treatment for immune‐related colitis is to give systemic high‐dose glucocorticoids, which have an efficacy of nearly 90% [29]. Early recognition of manifestations and timely interventions are necessary to lower the death risk in clinical treatment. In the updated guidelines for the systemic treatment of melanoma, although ipilimumab is no longer recommended for routine use as adjuvant therapy, the combination of ipilimumab plus nivolumab is still recommended for patients with metastatic cutaneous melanoma with particular mutations [30]. Therefore, fully understanding the potential death probability of each agent and for each organ system may provide practical treatment guidance. We found four deaths from nivolumab and atezolizumab that were related to the pulmonary system. Nivolumab‐related pneumonitis is a potentially life‐threatening AE [31]. A previous study indicated that severe cases were commonly seen in current and former smokers and in patients with underlying lung conditions [32]. Another notable lethal outcome in ICI adjuvant therapy is multiple organ failure. As a result of a series of complex causes, cases of multiple organ injury induced by cytokine release syndrome (CRS) have been occasionally reported in patients who received anti‐PD‐1 therapy [33, 34]. CRS is an uncontrolled systemic inflammatory response that can be triggered by certain drugs, and then progresses to multiple organ failure [35]. For clinical vigilance, fever should be considered as a sign of impending CRS for patients receiving T cell‐engaging therapies [36, 37]. Additionally, the susceptibility to TRAEs and IRAEs might be different in patients treated with ICI adjuvant therapy than in those with metastatic cancer. A previous study examined the causes of treatment‐related deaths from PD‐1 and PD‐L1 inhibitors and found that 8.5% of patients died from septic infections [7]. However, no deaths due to infection were found in our study. One possible explanation is that the tumor immune microenvironment has been demonstrated to be different across cancer stages: early, locally advanced, and advanced/metastatic. Braun et al. found an enrichment of terminally exhausted CD8+T cells and suppressive M2‐like macrophages in advanced and metastatic clear cell renal cell carcinoma, suggesting that immune dysfunction and inhibitory pathways play a critical role in cancer progression and response to immunotherapy [38]. The suppressed systemic immune status in patients with metastatic disease can alter the incidence of TRAEs from ICI treatments.

The safety profiles indicated that atezolizumab had a lower risk of causing grade ≥3 IRAEs than pembrolizumab. Unlike PD‐1 receptor, which is present on T cells, PD‐L1 ligands are expressed on the surface of tumor cells, in which the targets of ICIs differ. Inhibition of PD‐L1 may maintain some level of checkpoint signaling, and thus leads to onset of severe IRAEs less frequently. Although the results showed ipilimumab had a higher rate of treatment‐related death than pembrolizumab and nivolumab, we found that some organ toxicity indicators were lower in the former (hepatic, gastrointestinal, and endocrine). In other words, pembrolizumab and nivolumab could be safer agents than ipilimumab if the potential AEs are well managed. Here, we also comprehensively present the TRAEs and IRAEs that should be considered when giving each of the ICIs as adjuvant monotherapy.

– **Atezolizumab**: hyperthyroidism, hypothyroidism, pneumonitis, and hepatitis
– **Ipilimumab**: colitis, adrenal insufficiency, and hypophysitis
– **Nivolumab**: hyperthyroidism and hypothyroidism
– **Pembrolizumab**: hyperthyroidism, adrenal insufficiency, and diabetes mellitus

Compared with previous meta‐analyses of ICI therapies [6, 7], our network meta‐analysis has several strengths. To date, no study has comprehensively described the safety profile of adjuvant ICI monotherapies in pan‐cancer. Previous meta‐analyses have reported on PD‐1, PD‐L1, and CTLA‐4 inhibitors in combination therapies or in patients with advanced‐ and metastatic‐staged disease. This study combined incidence analysis and network meta‐analysis to optimize data accuracy. Deaths and AEs of all‐causes and as categorized by organ systems were analyzed, and some AEs of special interest were reported exclusively. Furthermore, to avoid any risks of bias in quality control and data collection, we only included phase III CRTs. We managed to construct a network that showed the difference between ipilimumab, atezolizumab, pembrolizumab, and nivolumab, the four ICIs that clinicians and investigators are most concerned with.

Several limitations of our study should be stated. First, moderate heterogeneity existed in the analysis of TRAEs, which could be related to the different numbers of patients enrolled in the 10 studies. Second, one study was enrolled despite being a conference abstract (KEYNOTE‐091), and the results may change when further research is completed. Furthermore, the range of start year of these enrolled studies spanned over 10 years; thus, the recognition and management of severe AEs might differ owing to varying facilities and experiences. Finally, several ongoing studies are investigating ICIs in the adjuvant setting; thus, updated results may enhance the reliability of our study.

## 5 CONCLUSIONS

In summary, ICI adjuvant therapies showed different safety in terms of deaths and AEs. Ipilimumab and atezolizumab were associated with a higher treatment‐related death rate than pembrolizumab and nivolumab, in which the gastrointestinal and pulmonary systems were mostly often involved, which is different from those with advanced or metastatic disease. Nivolumab and atezolizumab were safe with regard to severe TRAEs and IRAEs, respectively. These findings can guide clinical practice and optimize future trial designs for investigations of ICI adjuvant therapies.

## AUTHOR CONTRIBUTIONS

**Ruiyang Xie**: Data curation (equal); formal analysis (equal). **Jie Wu**: Data curation (equal); formal analysis (equal). **Bingqing Shang**: Investigation (equal); methodology (equal). **Xingang Bi**: Funding acquisition (equal); investigation (equal). **Chuanzhen Cao**: Conceptualization (equal); methodology (equal). **Youyan Guan**: Investigation (equal); project administration (equal). **Hongzhe Shi**: Funding acquisition (equal); investigation (equal). **Jianzhong Shou**: Project administration (lead).

## CONFLICT OF INTEREST

The authors declare no conflict of interest.

## ETHICS STATEMENT

The protocol was registered in the Prospective Register of Systematic Reviews (PROSPERO CRD42022339062).

## INFORMED CONSENT

Not applicable.

## Supporting information

Supplementary information.

## Data Availability

The datasets supporting the conclusions of this article are included within the article and its additional file.
